# Supplementary figures and images for: Conserved and cell type-specific transcriptional responses to IFN-γ in the ventral midbrain
Source: Brain Behav Immun. Author manuscript; Available in PMC 2023 Aug 27. (PMC10460506; doi:10.1016/j.bbi.2023.04.008)

# NeuN-

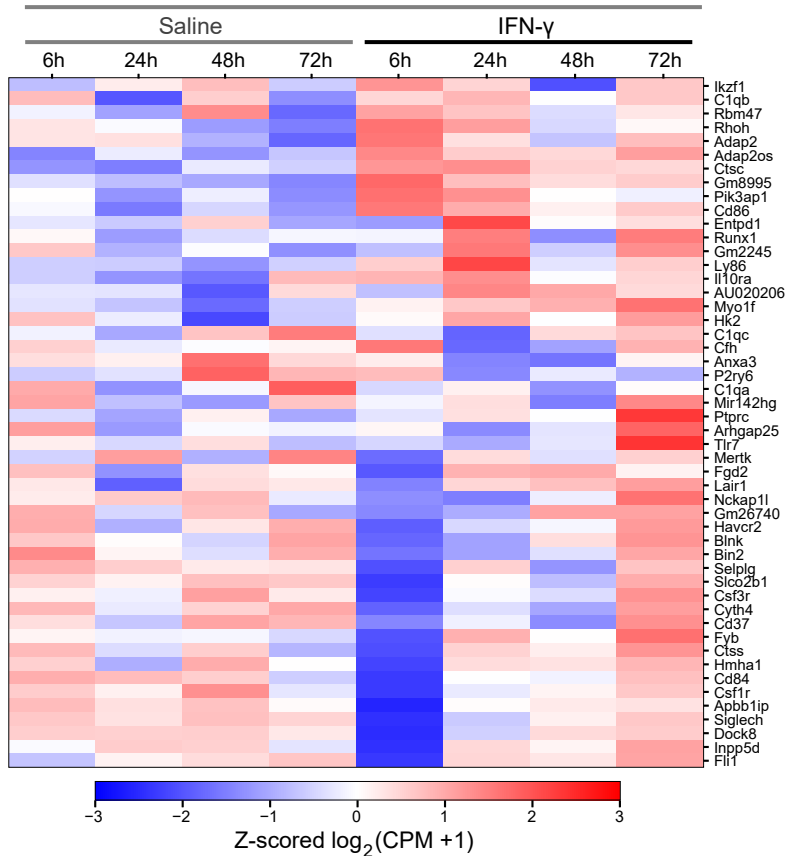

Supplement: Supp.Fig3 [file NIHMS1900921-supplement-Supp_Fig3.pdf]

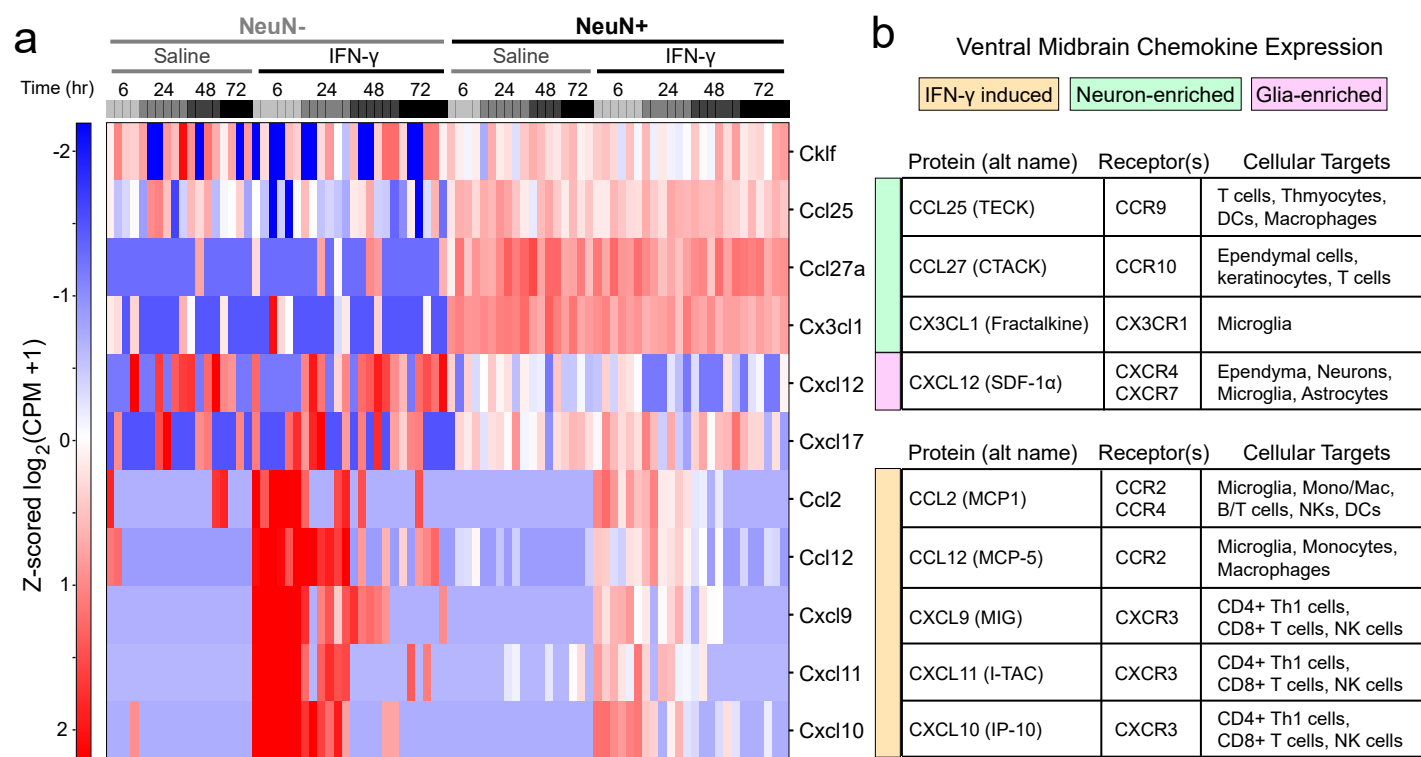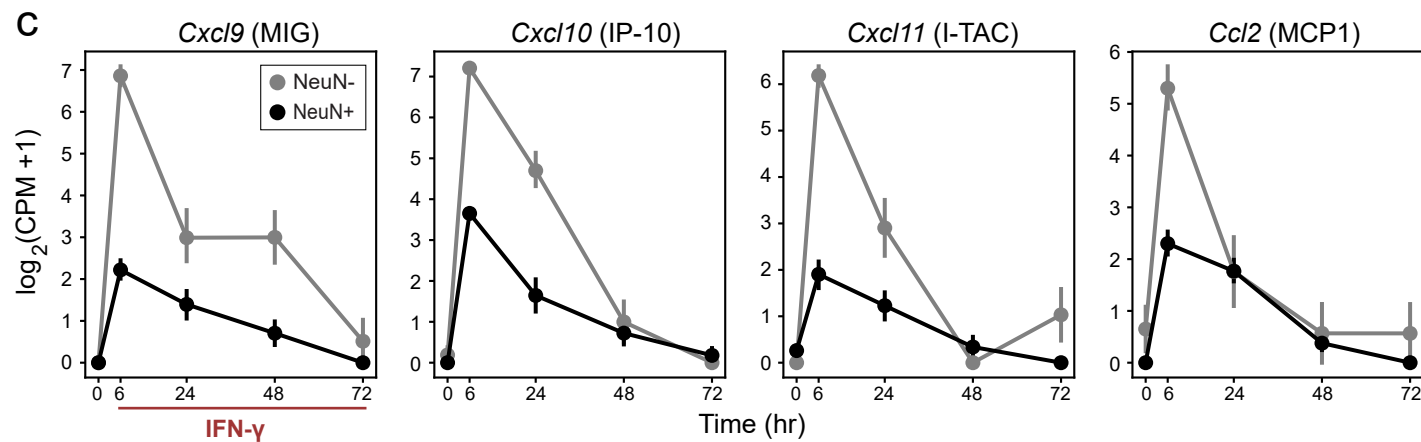

Supplement: Supp.Fig2 [file NIHMS1900921-supplement-Supp_Fig2.pdf]

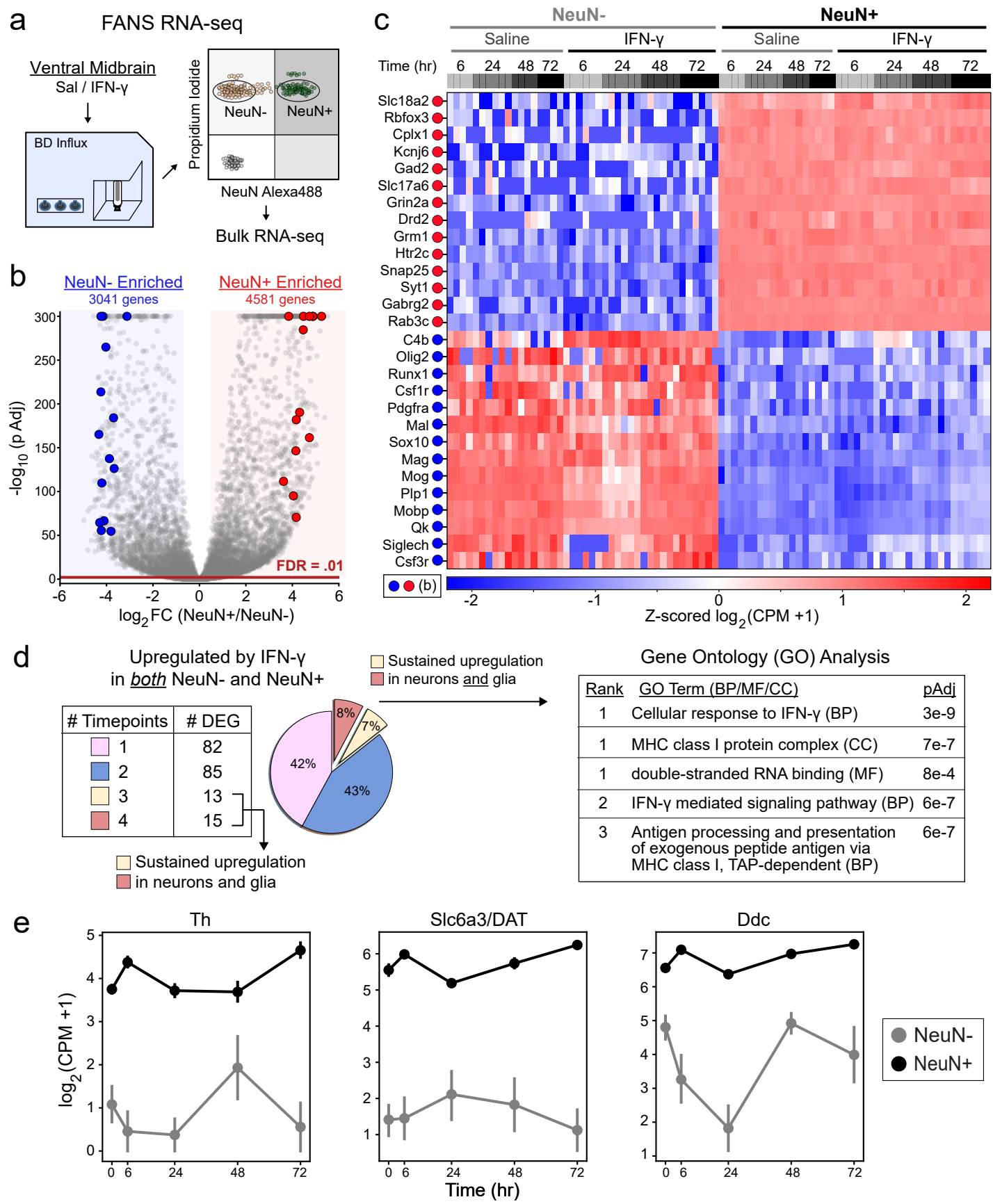

Supplement: Supp.Fig1 [file NIHMS1900921-supplement-Supp_Fig1.pdf]

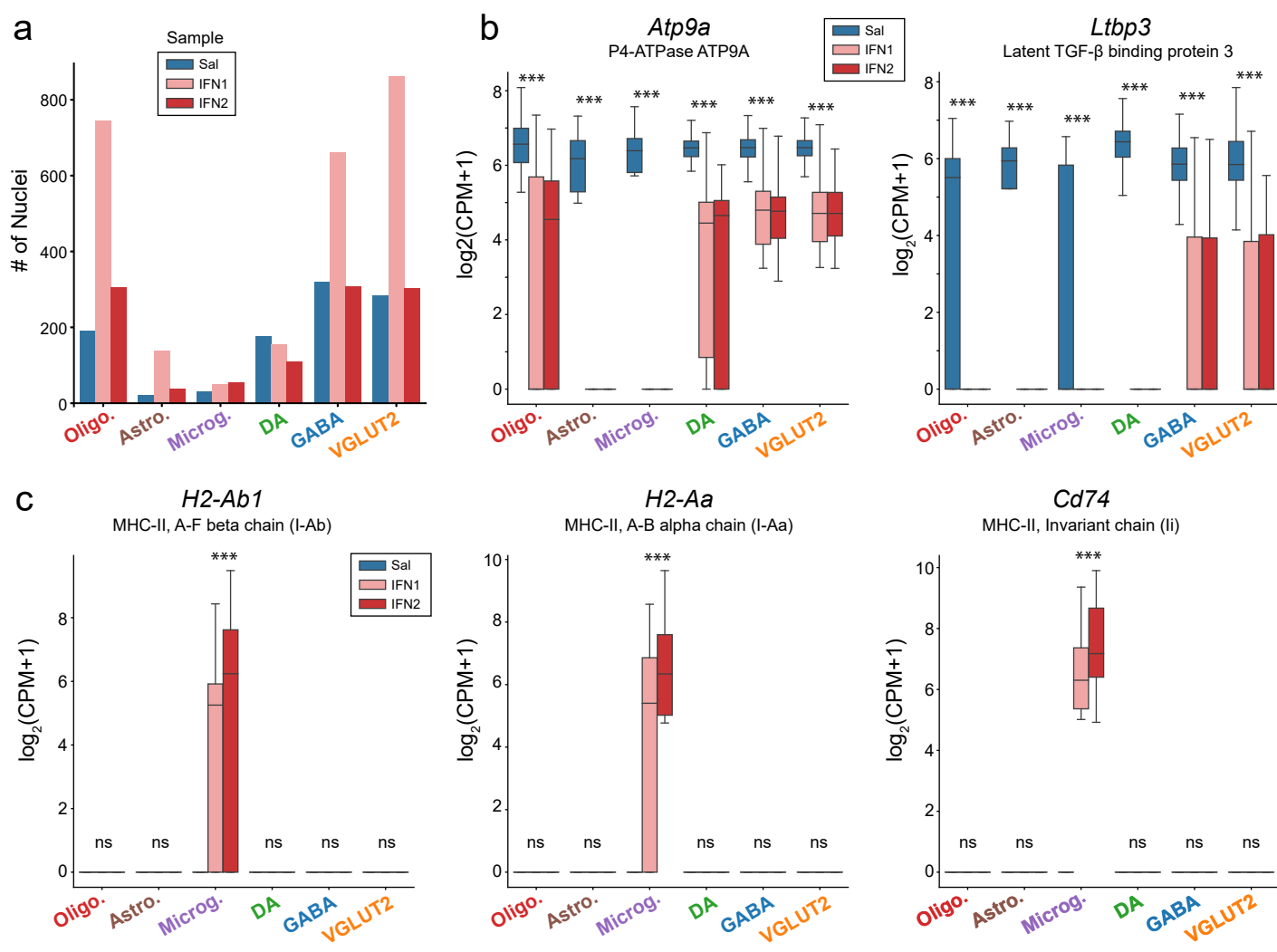

Supplement: Supp.Fig5 [file NIHMS1900921-supplement-Supp_Fig5.pdf]

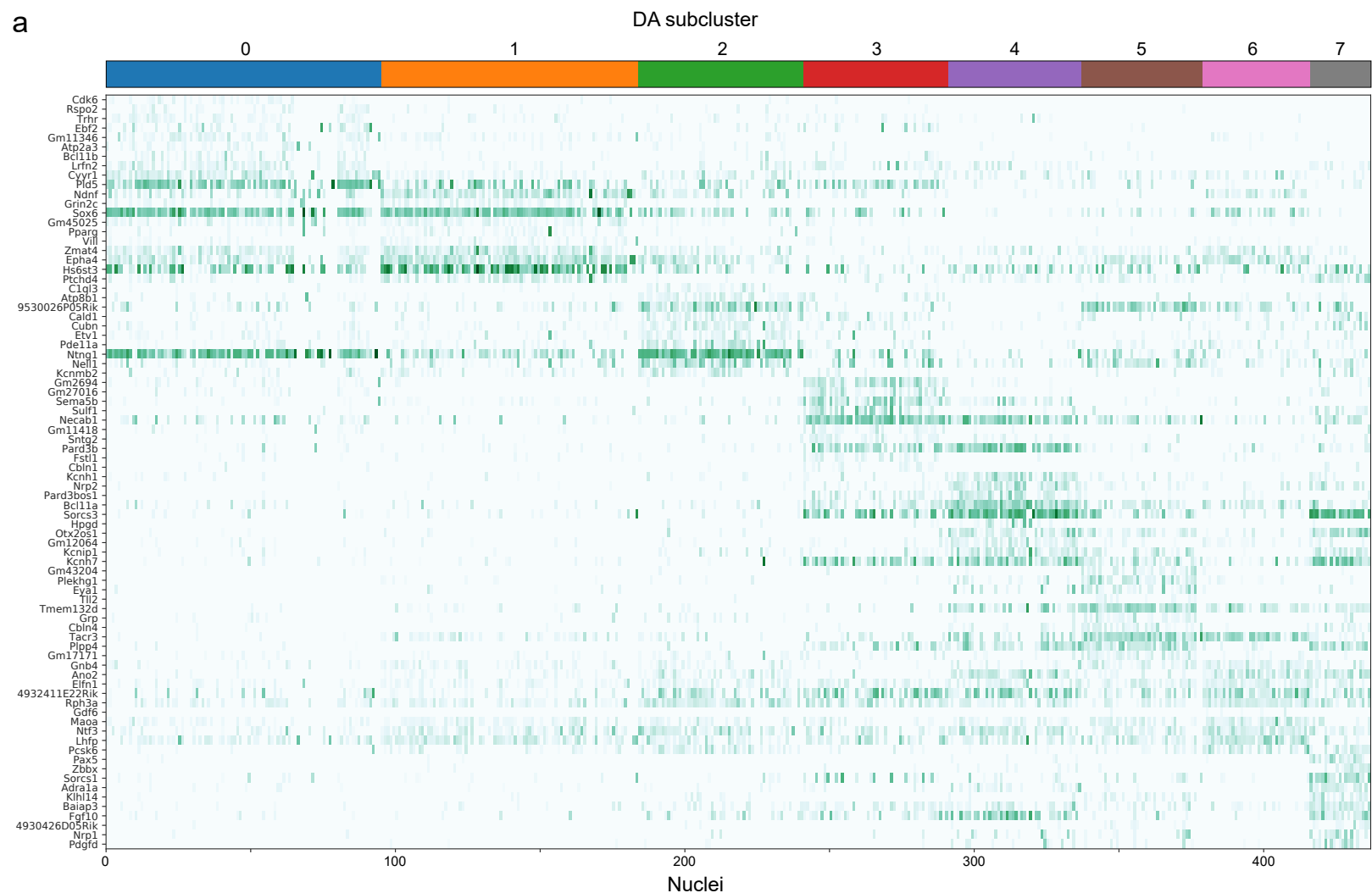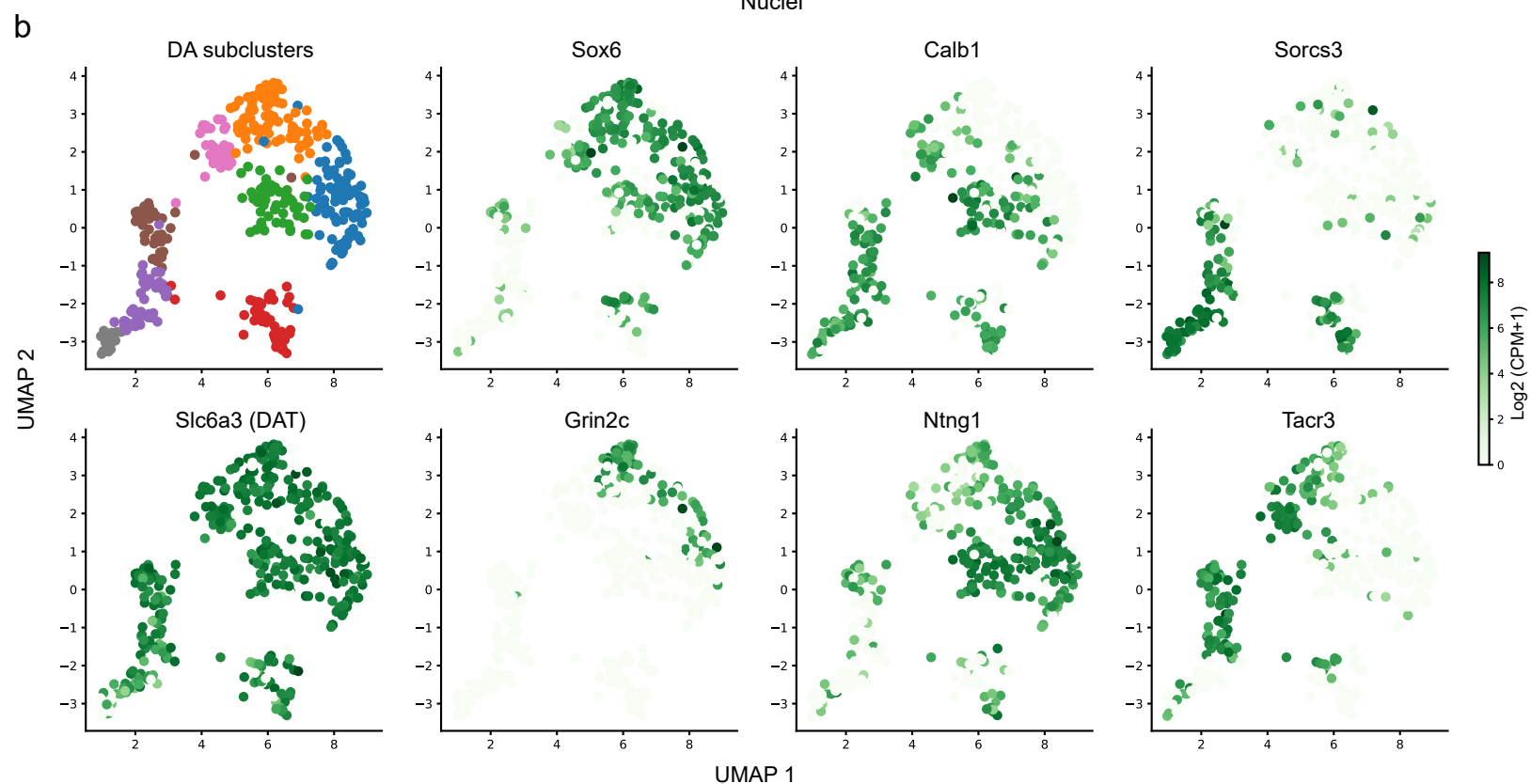

Supplement: Supp.Fig7 [file NIHMS1900921-supplement-Supp_Fig7.pdf]

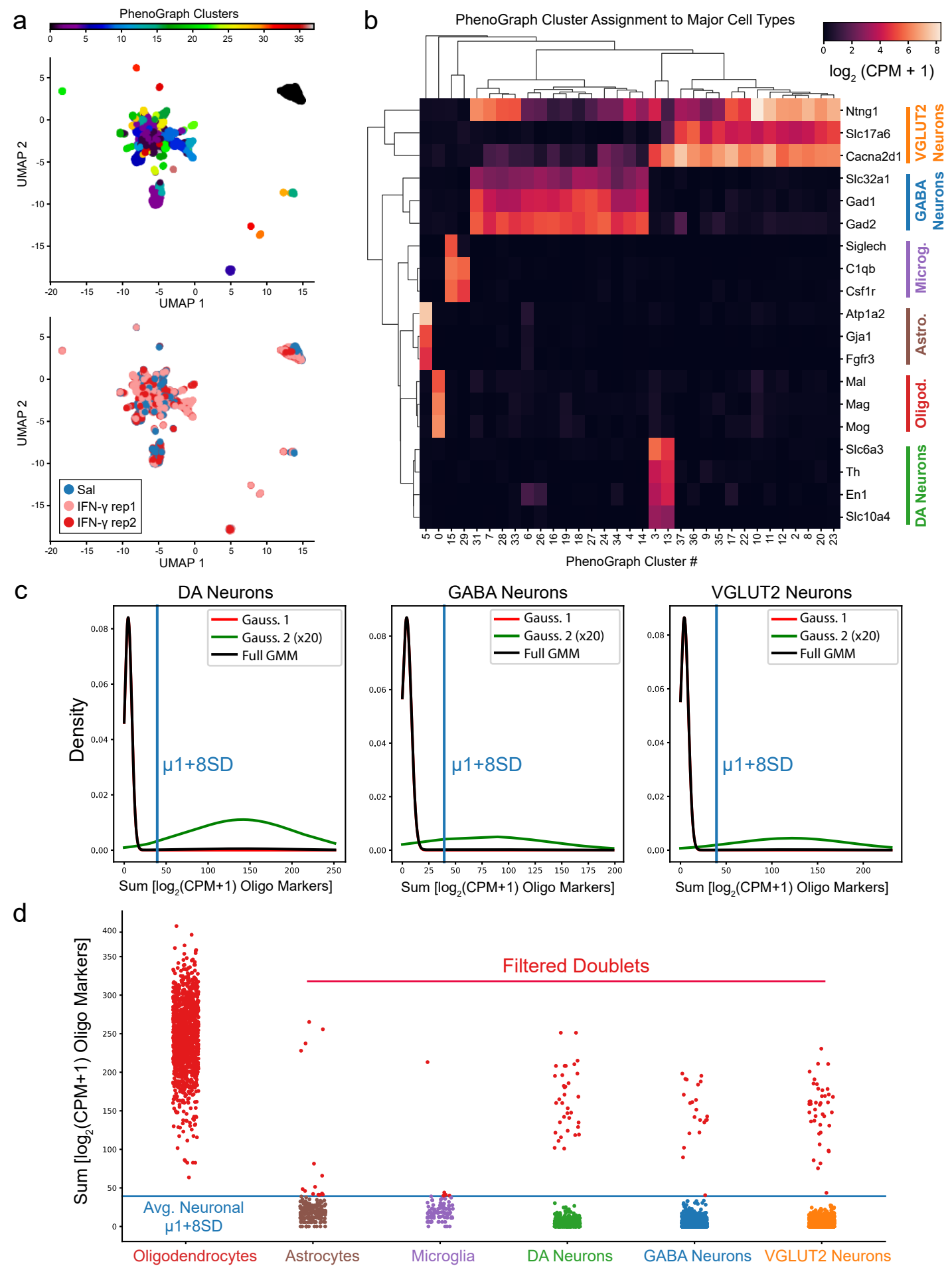

Supplement: Supp.Fig4 [file NIHMS1900921-supplement-Supp_Fig4.pdf]

a

TH IF    *Ifngr1* RNA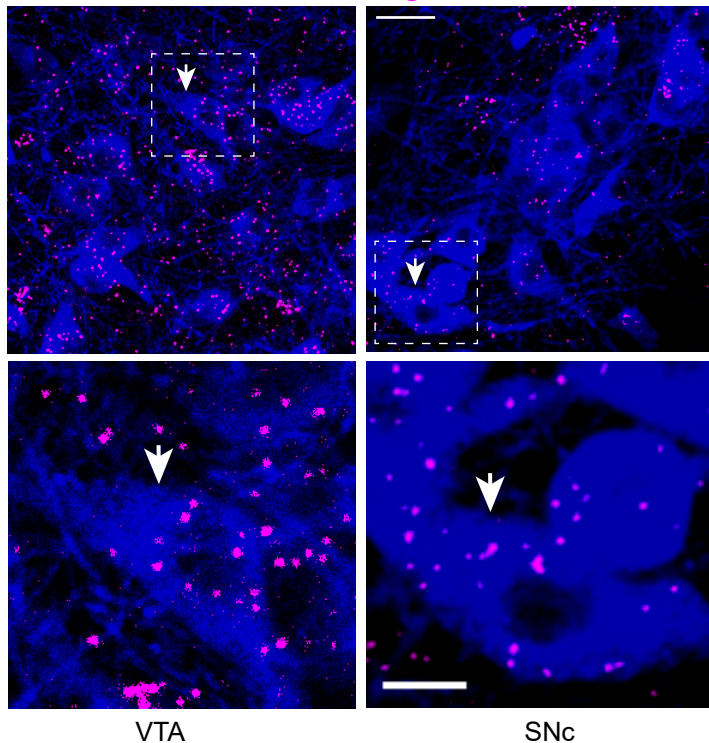

b

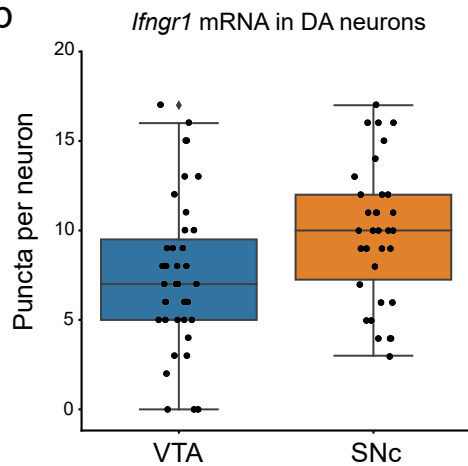

Supplement: Supp.Fig8 [file NIHMS1900921-supplement-Supp_Fig8.pdf]

a

Sal

IFN

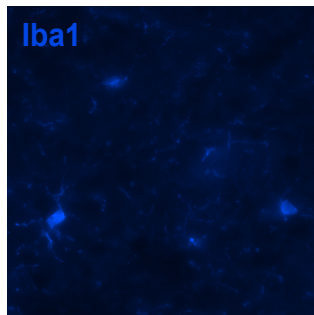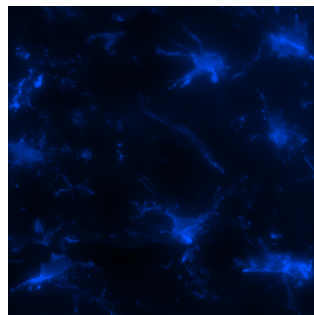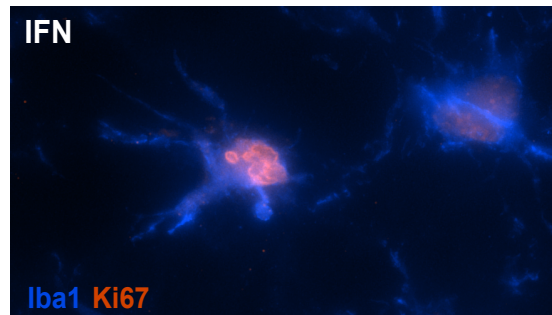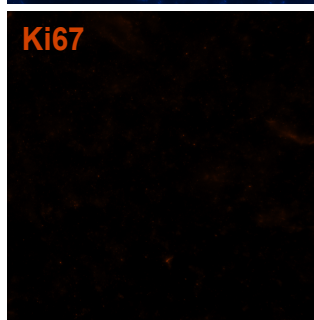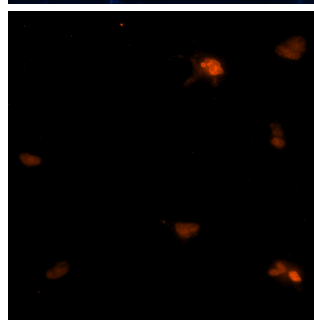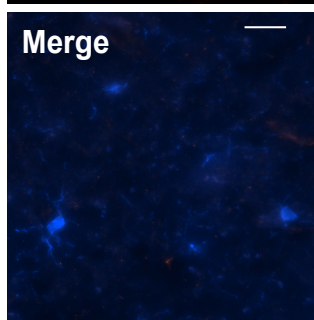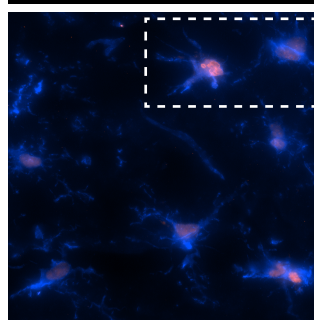

b

IFN-induced Microglial Proliferation

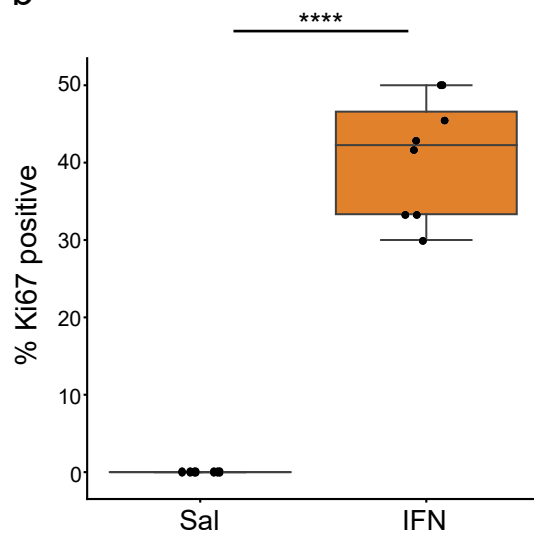

Supplement: Supp.Fig6 [file NIHMS1900921-supplement-Supp_Fig6.pdf]
