# Supplementary material for: Conserved and cell type-specific transcriptional responses to IFN-γ in the ventral midbrain
Source: Brain Behav Immun. Author manuscript; Available in PMC 2023 Aug 27. (PMC10460506; doi:10.1016/j.bbi.2023.04.008)
Supplement: Supp.Fig9 [file NIHMS1900921-supplement-Supp_Fig9.pdf]

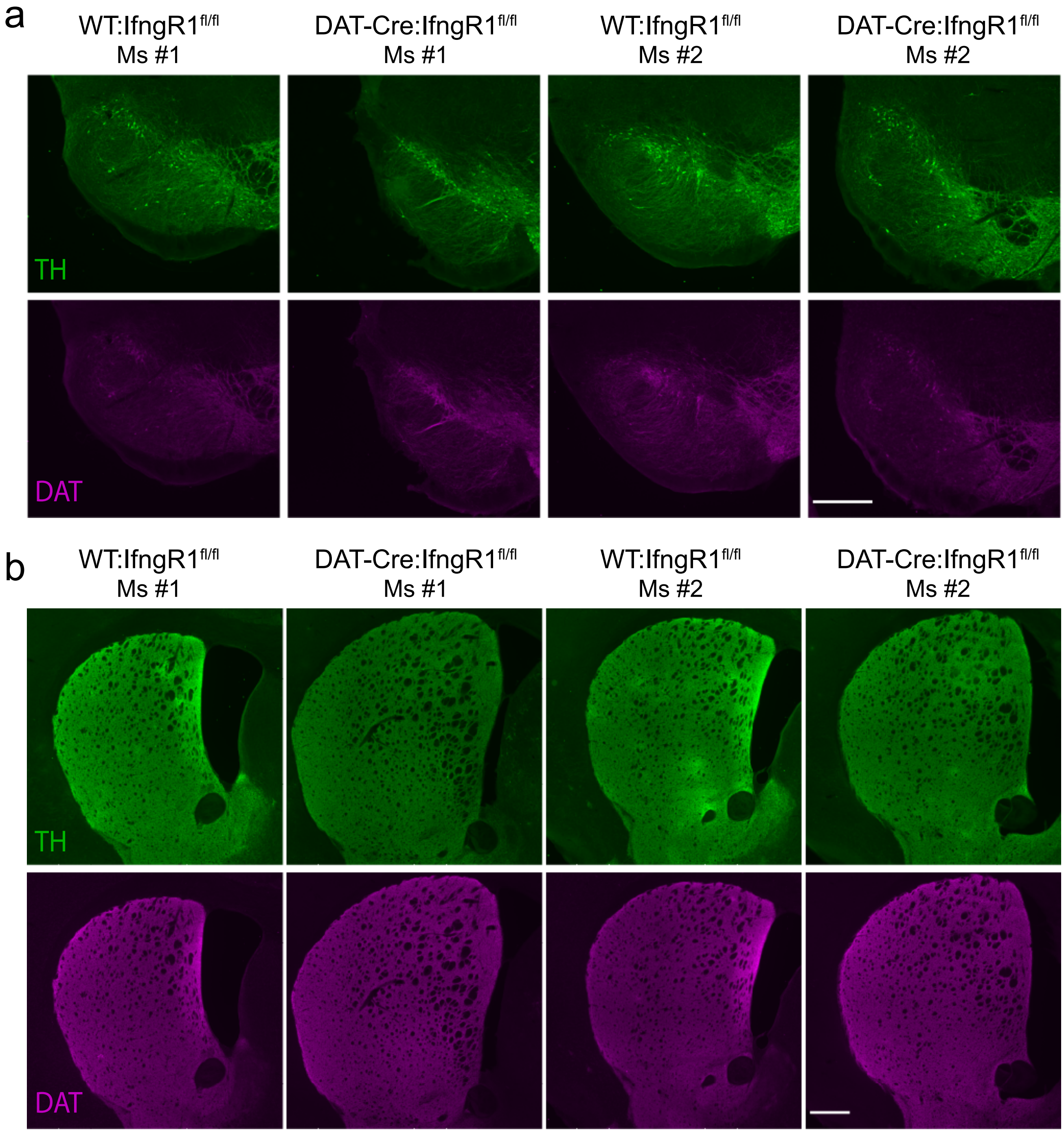

a
WT:IfngR1fl/fl Ms #1
DAT-Cre:IfngR1fl/fl Ms #1
WT:IfngR1fl/fl Ms #2
DAT-Cre:IfngR1fl/fl Ms #2
TH
DAT
b
WT:IfngR1fl/fl Ms #1
DAT-Cre:IfngR1fl/fl Ms #1
WT:IfngR1fl/fl Ms #2
DAT-Cre:IfngR1fl/fl Ms #2
TH
DAT
